# Accuracy of site benchmarking in clinical quality registries of varying size

**DOI:** 10.1177/18333583251355820

**Published:** 2025-07-23

**Authors:** Jessy Hansen, Ahmad Reza Pourghaderi, Susannah Ahern, Arul Earnest

**Affiliations:** School of Public Health and Preventive Medicine, Monash University, Melbourne, VIC, Australia

**Keywords:** benchmarking, data analysis, registries, clinical quality registries, health information management

## Abstract

**Background:** There is increasing interest in the public reporting of health provider benchmarking within clinical registries to identify underperforming sites (also known as outliers). As such, research into the optimal methods and ideal conditions for outlier detection is important. **Objective:** The aim of this study was to assess the accuracy of benchmarking and outlier classification methods for different values of clinical registry sizes and case volume minimums. **Method:** Clinical registry datasets were parametrically simulated varying the following parameters: number of sites, clinicians, patients and outcome events, case volume minimum and outcome prevalence. Two benchmarking models (unadjusted and risk-adjusted with logistic regression) and two outlier classification techniques (confidence intervals and control limits) were applied to each simulated dataset. The accuracy of outlier flagging was assessed using the receiver operator characteristic area under the curve (ROCAUC). **Results:** Risk-adjusted benchmarking performed better than unadjusted benchmarking across the registry sizes evaluated, providing up to a 20% increase in ROCAUC. The number of sites and clinicians had little effect on performance, while higher accuracy with increasing number of patients per site and outcome prevalence was observed. A threshold of 100 to 150 outcome events per site was needed to reach >80% ROCAUC. **Conclusion:** The use of low prevalence outcomes for benchmarking hospitals to detect outliers may be inappropriate, especially for clinical registries with low patient volumes. **Implications for health information management practice:** Clinical registries should consider their patient volumes and outcome prevalence before commencing benchmarking analyses to determine if acceptable accuracy can be achieved for their setting.

## Introduction

Clinical quality registries (CQRs) are repositories of prospectively collected health data that can provide valuable insight into patient care, quality of treatment and outcomes (Hoque et al., 2017; Stey et al., 2015). Registry establishment is on the rise worldwide (Australian Commission on Quality and Safety in Health Care (ACSQHC), 2022a; Blumenthal, 2017; Hoque et al., 2017), reporting on a range of patient populations, including those recently diagnosed with a specific disease (such as cancer registries), those undergoing a certain procedure type or receiving a medical device (such as surgical and device registries) and patients who experienced a significant health event (such as stroke or trauma) ((ACSQHC), 2022a; Hoque et al., 2018). CQRs collect specified clinical data that are often lacking in routinely collected datasets (like medical records), making them ideal for health monitoring and research (Lin et al., 2020; Liu et al., 2017; Psoter and Rosenfeld, 2013).

A key purpose of CQRs is to track patient treatment and outcomes to drive quality improvement, often through the benchmarking of health providers (such as hospital sites or clinicians) to detect underperformers, known as outliers (Brown et al., 2022; Hoque et al., 2017; McNeil et al., 2010; Wilcox and McNeil, 2016). Benchmarking is the comparison of provider performance on clinical indicators, which can range from process indicators (like antibiotic use) to outcomes (such as mortality) (ACSQHC, 2022b; Department of Health, 2021; Evans et al., 2011). The outcome rate for each provider is calculated, often using risk-adjustment to control for patient risk factors, and then compared to a “benchmark,” usually the population mean or median. Statistical methods are then applied to determine providers that deviate from expected performance (outliers); the identified providers can then be notified, monitored, and targeted for performance improvement (Department of Health, 2021; Hoque et al., 2017; Wilcox and McNeil, 2016).

Governments are often involved in such activities, including in Australia where the ACSQHC (2022b) has listed the benchmarking of providers and outlier management as a required output for registries.. Further, there is increasing interest in the public reporting of outliers to inform patients and stakeholders (Ahern et al., 2017; Behrendt and Groene, 2016), making accurate methods of provider benchmarking and outlier detection vital. False positives, caused by the incorrect flagging of providers performing within the expected range, could result in undue reputational or financial repercussions for those incorrectly classified, especially if publicly reported. On the other hand, false negatives can contribute to poorer patient treatment and outcomes as underperforming providers will remain undetected.

Much research has investigated the use of provider benchmarking methods with administrative data, such as routinely collected medical and insurance records (Eijkenaar and van Vliet, 2014; Goldstein and Spiegelhalter, 1996; Paddock, 2014; Spiegelhalter et al., 2012); however, there is little consensus on the optimal method. In addition, many studies have found the accuracy of outlier detection to vary across different data settings, with poor accuracy observed for outcomes of low prevalence (such as mortality) and analyses with low case/patient volumes (Hofer and Hayward, 1996; Seaton et al., 2013; Thomas and Hofer, 1999; Walker et al., 2013). Further, little research has evaluated the accuracy of common benchmarking and outlier classification methods in the context of CQRs (Hansen et al., 2023), and there is a lack of guidance available on the optimal methods for different registry settings. A recent statistical simulation study conducted by the authors found the accuracy of commonly used benchmarking and outlier classification techniques (ordinary, fixed, and random-effects logistic regression models, and different confidence interval and control limit calculations) to vary between methods and across different outcome parameters (Hansen et al., 2025). Poor accuracy in particular was found for random effects regression, and outliers flagged for outcomes of extreme prevalence (low and high), high dispersion and poor risk-adjustment fit.

Following on from the previous work, this study aimed to evaluate the accuracy of commonly used benchmarking and site level outlier detection methods over a range of registry sizes (number of sites, clinicians, patients and outcome events), case volume minimums, and outcome prevalence through the use of a parametric simulation study. Rather than provide novel methodological findings, this study aimed to provide specific insights for the application of outlier classification methods when benchmarking sites in CQRs, including recommendations for what registry size is needed for accurate analyses and whether case volume minimums for determining provider inclusion in benchmarking are appropriate.

## Method

The simulation study design was developed to the ADEMP (aims, data-generating mechanisms, estimand/target, methods and performance measures) framework (Morris et al., 2019), details of which can be found in Supplemental File S1. The Stata program developed to simulate the data, apply the methods and store the performance measures is available online in the Monash Bridges repository.^1^ All analyses were performed with Stata/SE V17. The datasets generated and analysed for the current study are available in the Monash Bridges repository.^2^ Briefly, hierarchical three-level datasets were generated to reflect the clustered structure of CQR data, with patients, nested within clinicians, nested within sites. The total number of patients, clinicians and sites in each simulation were specified; the number of patients assigned to each clinician, and clinicians assigned to each site were randomly allocated using a Gamma distribution. Sites were randomly assigned outlier status (to provide a known “true” outlier state for evaluating classification accuracy) using a binomial distribution with *p* = 0.05. A logit model was used to parametrically simulate patient-level outcome risk, p_ijk_ (outcome), with which patient outcomes were randomly generated from a binomial distribution. The logit model included terms for specifying outcome prevalence (with an additional term for patients from “true” outlier sites to provide an underlying outcome prevalence of two times that of patients from non-outlier sites), random variance at the patient, clinician and site levels, and a continuous risk-factor variable (to emulate a risk-score for use in risk-adjustment). The risk-factor values for each patient were randomly assigned from a normal distribution with a sample variance sufficient to give a “good” model fit of ~80% AUC. Sites with a patient number (case volume) below the case volume minimum were excluded from benchmarking analyses. Full details of the data-generating mechanisms and simulation model, terms and calculations can be found in Online Supplemental File S1, including equation (1) (logit model for patient risk, p_ijk_(outcome)) and equation (2) (binomial outcome based on patient risk), and Supplemental Table S1.

The simulations presented in this study varied the parameters of the number of sites, number of clinicians per site, number of patients per clinician and case volume minimum. The parameter values and combinations evaluated in the study are outlined in Box 1. Parameter values were chosen to cover common values and extend to more extreme values, with the selection guided by a recent systematic review and the CQRs present in the Australian Register of Clinical Registries (ACSQHC, 2022a; Hansen et al., 2023). All registry size parameter combinations were evaluated at three values of outcome prevalence (patient level average), 5%, 40% and 90% (representing low, mid and high values, respectively), to provide an interaction analysis given the known impact of outcome prevalence on accuracy. The parameters of patients per site and outcome events per site were additionally evaluated with data from the simulations varying the number of patients, clinicians and sites. For each unique combination of parameter values (scenario), 1000 datasets were generated to which commonly used benchmarking and outlier detection methods were then applied. The evaluated methods were chosen based on the best performing techniques assessed in a previous simulation study: two methods of generating site rate estimates (unadjusted and risk-adjusted with logistic regression) and two outlier classification techniques (95% exact binary control limits and 95% Byar approximation confidence intervals). Control limit methods are data-independent, with limits of allowable deviation from the benchmark calculated for a range of case volumes (using the exact binomial calculation for this study); sites whose outcome prevalence is outside the limit are then flagged as outliers. Confidence intervals are data-dependent methods, with confidence bounds calculated for individual site rate estimates (using the Byar approximation to the exact Poisson calculation for this study); sites whose lower confidence bound is above the benchmark are flagged as outliers. Details of the regression models and classification technique calculations can be found in Supplemental Tables S2 and S3, Online Supplemental File S1.

**Box 1.** Registry parameters varied in the simulation, with a summary of investigated values and parameter combinations.

Parameter	Values ^a^	Combinations with other parameters ^b^
Number of sites	5, 10, 25, 50, 100, 250	Clinicians/site: 2, 10, 50 Patients/clinician: 10, 100, 250
Number of clinicians (per site)	2, 10, 50	Patients/clinician: 10, 100, 250
Number of patients (per clinician)	10, 100, 250	Clinicians/site: 2, 10, 50
Number of patients (per site)	From combinations of number of sites, clinicians and patients	Number of sites: 10, 25, 100
Number of events (per site)		
Case volume minimum	0, 10, 50, 150, 250	Patients/site: 250,500
Outcome prevalence	5%, 40%, 90%	All other combinations

a Default value underlined.

b Presents the factorial combinations with other parameters that were evaluated for each parameter. For example, for each value of case volume minimum, datasets were simulated with 250 and 500 patients/site.

The two site estimate methods and two outlier classification techniques gave a total of four method combinations that were applied to each simulated dataset. The ability of each method combination to accurately classify sites (compared to their “true” simulated outlier status) was assessed using the receiver operator characteristic area under the curve (ROC AUC), which represents the trade-off between sensitivity and specificity. The sensitivity, specificity and positive and negative predictive values were additionally calculated (details on the performance measures can be found in Supplemental Table S4, Online Supplemental File S1). The average performance across the 1000 simulations of each scenario and 95% confidence intervals were calculated to evaluate the performance measures for all method combinations.

### Ethics approval

Ethics approval was not required as this article does not contain any studies with human or animal participants.

## Results

The ability of the evaluated methods to correctly classify outlying sites varied both between methods and across different registry size parameters and case volume minimums. Due to the large number of parameter combinations evaluated, this section presents the ROC AUC results of varying each size parameter while holding the other factors constant at the default value (except for case volume minimum, which is presented for two values of patient per site) for all three values of outcome prevalence; complete results for all parameter combinations and additional performance measures can be found in Online Supplemental Figures S1 to S34, Online Supplemental File S2. The total number of sites, clinicians and patients for each scenario is available in Table S5, Online Supplemental File S2. Six parameter combinations were not possible to run due to unfeasible computation times, three for simulations with 100 sites and 5000 clinicians (with 50,000, 500,000, and 1,250,000 patients), two with 250 sites and 2500 clinicians (with 25,000 and 625,000 patients) and one with 250 sites, 12,500 clinicians and 1250000 patients.

### Models and outlier classification methods

**Figure 1.**
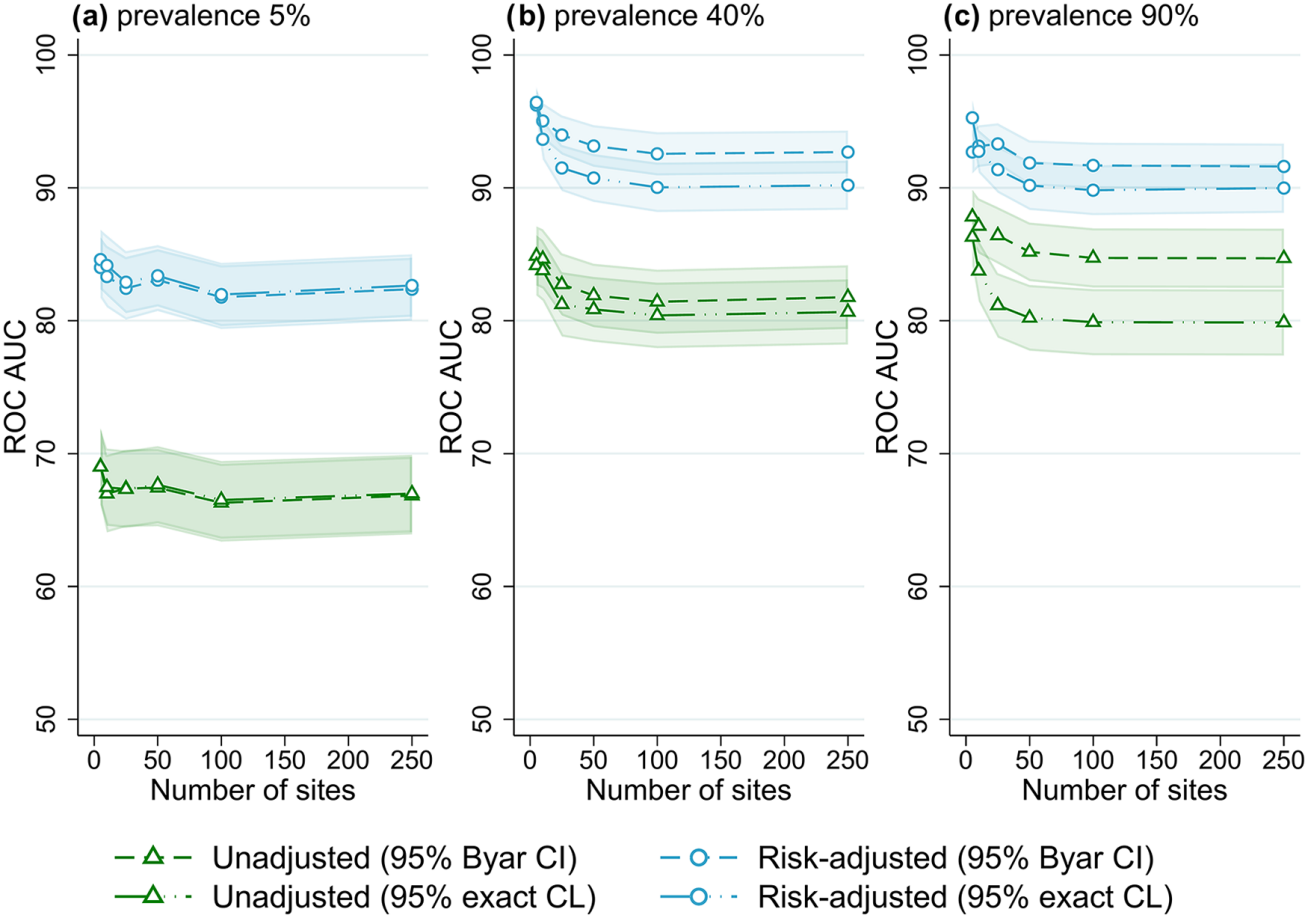
Receiver operator characteristic area under the curve (ROC AUC) (with 95% confidence intervals) for four outlier classification methods when varying the number of sites, for three outcome prevalence values, (a) 5%, (b) 40% and (c) 90%.

**Figure 2.**
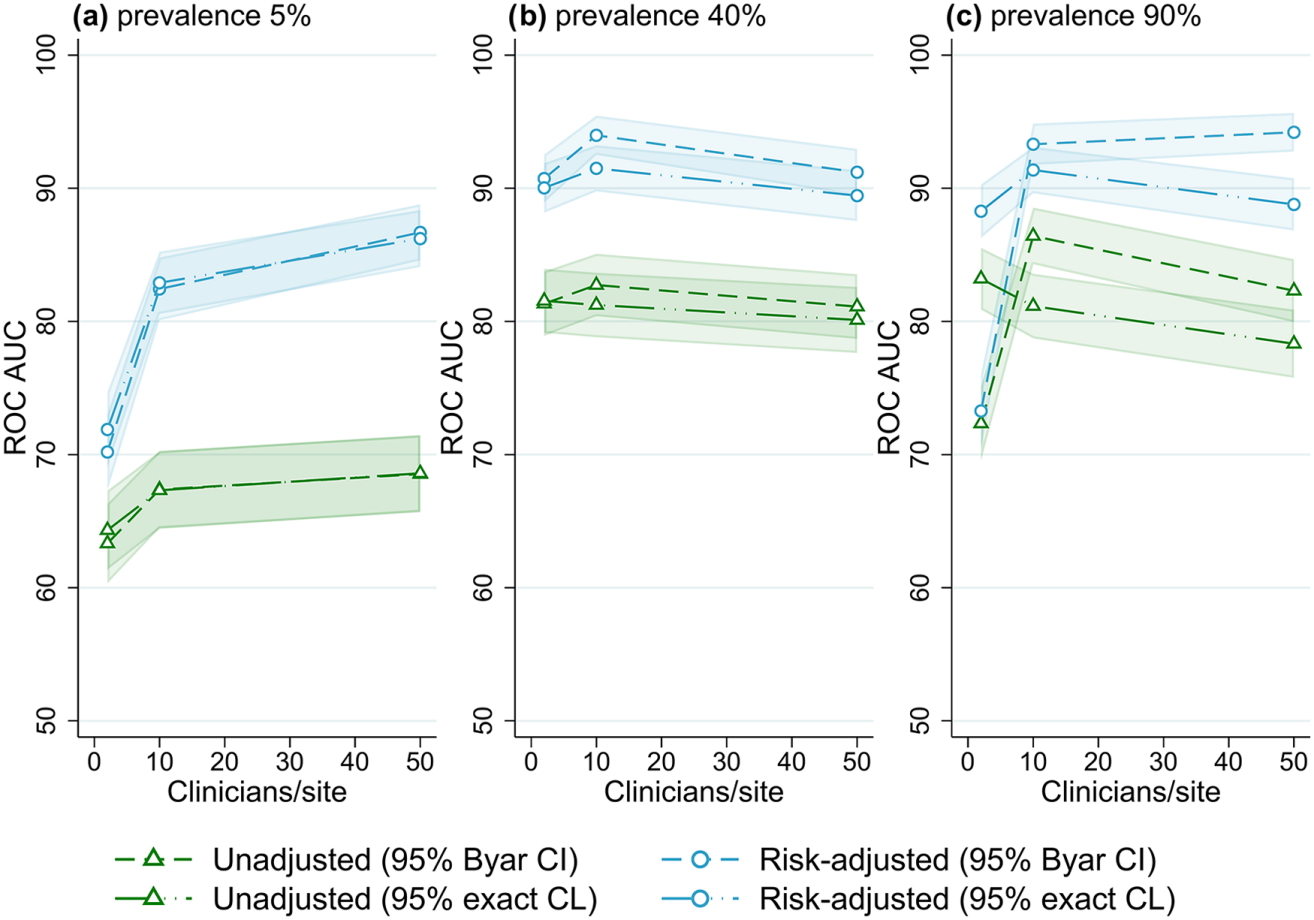
Receiver operator characteristic area under the curve (ROC AUC) (with 95% confidence intervals) for four outliers classification methods when varying the number of clinicians per site, for three outcome prevalence values, (a) 5%, (b) 40% and (c) 90%.

**Figure 3.**
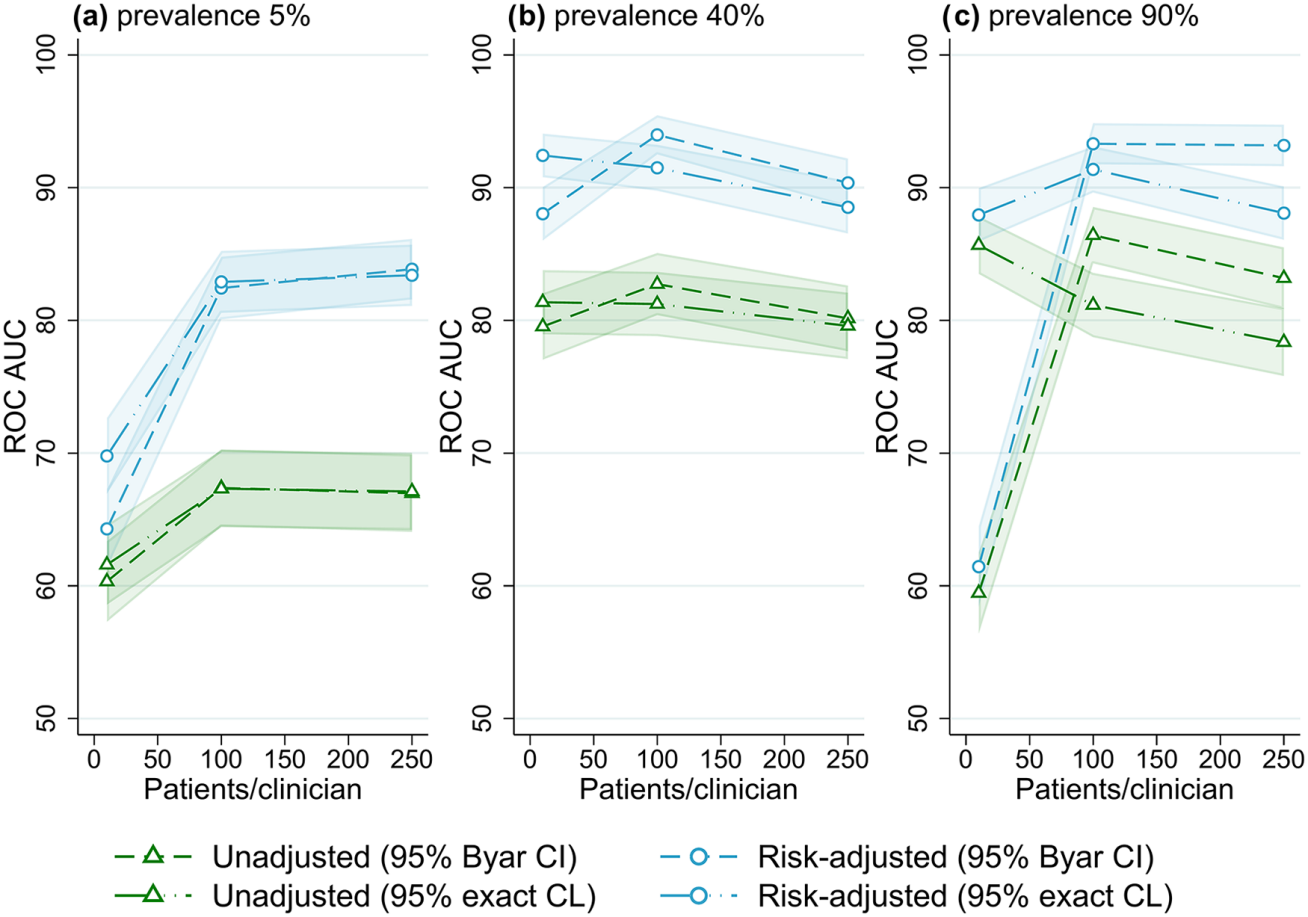
Receiver operator characteristic area under the curve (ROC AUC) (with 95% confidence intervals) for four outlier classification methods when varying the number of patients per clinician, for three outcome prevalence values, (a) 5%, (b) 40% and (c) 90%.

**Figure 4.**
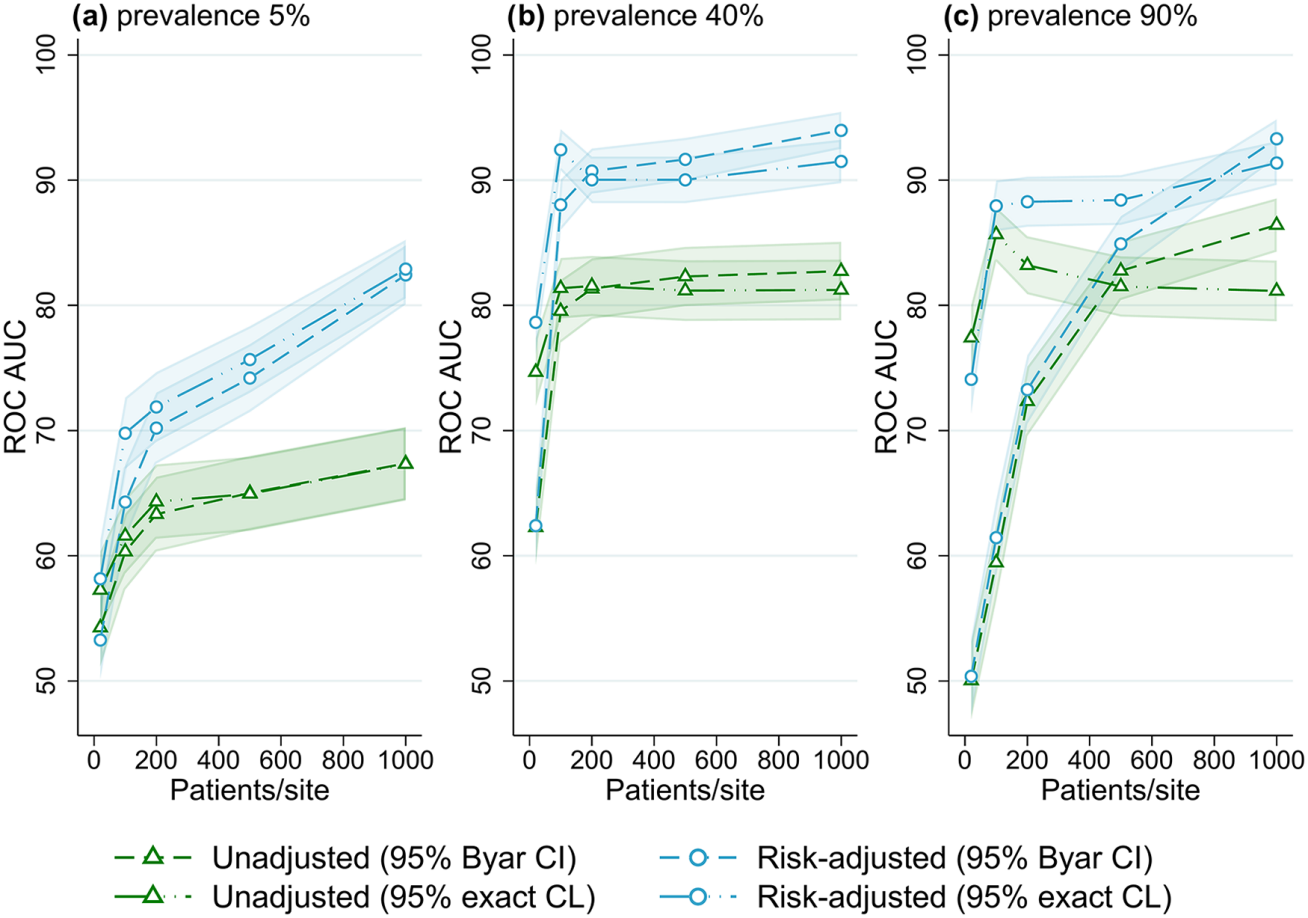
Receiver operator characteristic area under the curve (ROC AUC) performance (with 95% confidence intervals) for four outlier classification methods when varying the number of patients per site, for three outcome prevalence values, (a) 5%, (b) 40% and (c) 90%.

**Figure 5.**
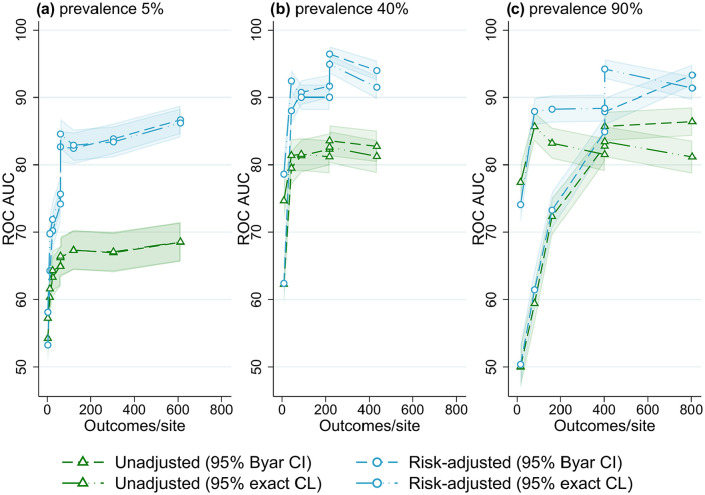
Receiver operator characteristic area under the curve (ROC AUC) performance (with 95% confidence intervals) for four outlier classification methods when varying outcome events per site, for three outcome prevalence values, (a) 5%, (b) 40% and (c) 90%.

**Figure 6.**
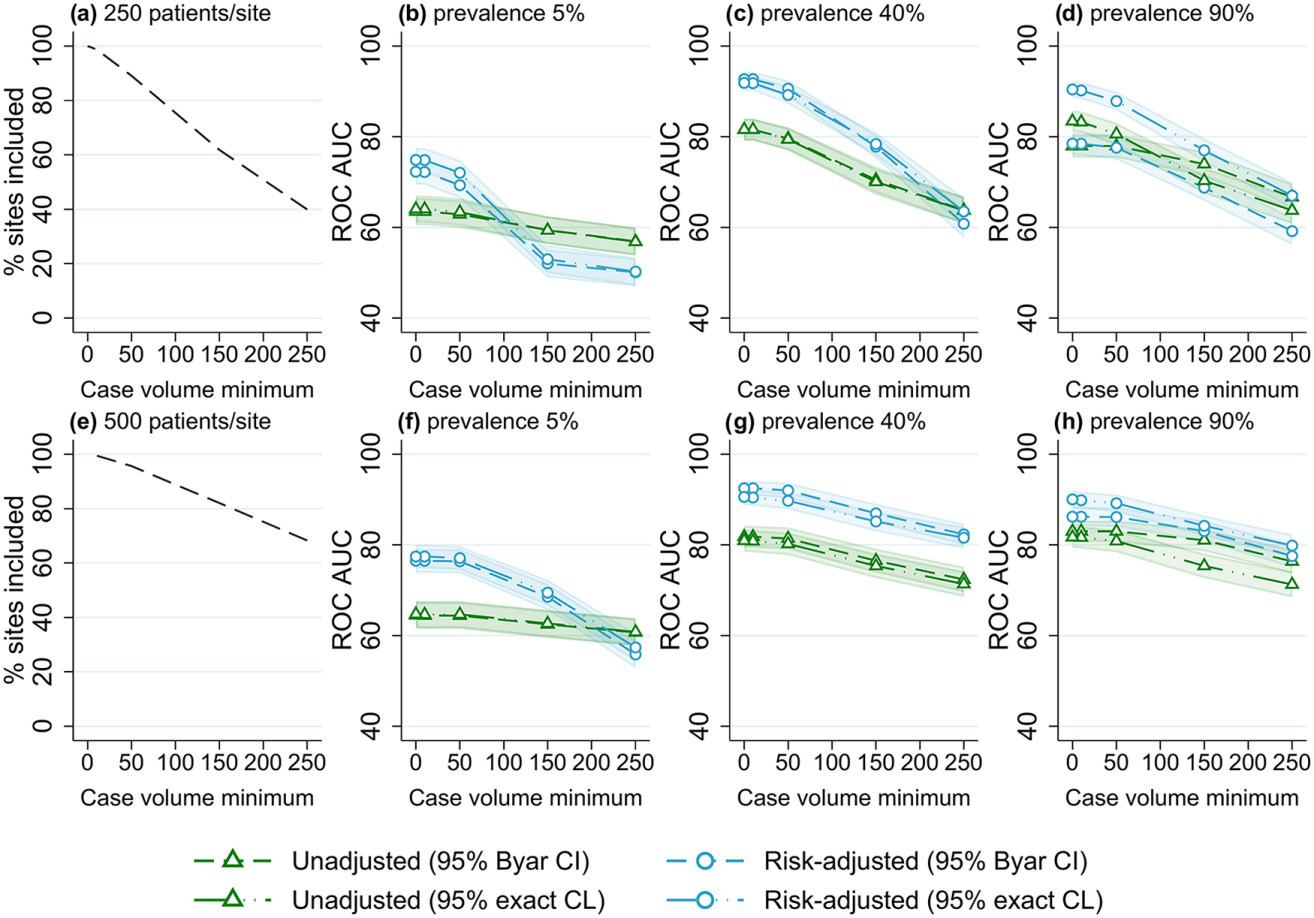
Proportion of included sites and receiver operator characteristic area under the curve (ROC AUC) (with 95% confidence intervals) for four outlier classification methods when varying the case volume minimum, for three outcome prevalence values (5%, 40% and 90%), and two numbers of patients per site, (a–d) 250 and (e–h) 500.

The best overall method combination was risk-adjusted site rates obtained from logistic regression with 95% exact binomial control limits used to classify outlying sites. For almost all scenarios evaluated, outlier detection with risk-adjusted estimates was more accurate than unadjusted site rates (Figures 1–6); benchmarking sites with unadjusted rates performed similarly to risk-adjusted analyses only in scenarios with low patient numbers, where all methods performed poorly. For the simulations varying the number of sites (Figure 1), at low-outcome prevalence risk-adjustment improved accuracy by over 10% ROC AUC (from <70% for unadjusted to >80% for adjusted); at mid- and high-outcome prevalence risk-adjustment improved the accuracy of outlier classification from ~80% ROC AUC for unadjusted to ~90% for risk-adjusted. Control limits were more accurate than confidence intervals in simulations with lower patient numbers, especially at high-outcome prevalence, and confidence intervals had marginally better performance in simulations with higher patient numbers and higher prevalence; performance was otherwise similar between the techniques (Figures 1-6).

### Number of sites

Figure 1 shows the performance of the evaluated benchmarking and outlier classification methods to accurately detect outliers while varying the number of sites for three different outcome prevalence values, for the default number of clinicians per site (10) and patients per clinician (100) (results for all combinations of clinician and patient numbers evaluated can be found in Supplemental Figures S1–S10, Online Supplemental File S2). There was little association between the number of sites and the performance of outlier classification, aside from a marginal decrease in accuracy (2-5% ROC AUC) when increasing from five to 25 sites. After this performance was stable, with an ROC AUC for risk-adjusted control limits of around 82% for low-outcome prevalence and 90% for mid- and high-outcome prevalence. Results were similar when evaluating different numbers of clinicians and patients, and other performance measures (Supplemental Figures S1–S10, Online Supplemental File S2).

### Number of clinicians

Figure 2 shows the overall performance of the methods for varied numbers of clinicians (relative to the number of sites) for different outcome prevalence, for the default number of sites (25) and patients per clinician (100) (results for all combinations of site and patient numbers evaluated can be found in Figures S11–S15, Online Supplemental File S2). At low-outcome prevalence, there was an increase in the accuracy of outlier detection with an increasing number of clinicians, from ~70% ROC AUC for two clinicians per site to approximately 85% for 50 clinicians per site (for risk-adjusted rates). The number of clinicians had little effect on outlier detection performance for mid- and high-outcome prevalence scenarios.

### Number of patients and events

The association between the number of patients per clinician and outlier identification performance for different outcome prevalence is shown in Figure 3. At low-outcome prevalence, the accuracy of flagging increased from ~70% ROC AUC for 10 patients per clinician to about 85% for 250 patients per clinician (for risk-adjusted rates). At higher-outcome prevalence, there was little association between the number of patients per clinician and outlier detection performance (except for very low ROC AUC for confidence intervals in scenarios with 90% outcome prevalence and low patient numbers).

Figure 4 shows the association between performance and the number of patients per site, at different levels of outcome prevalence. For the lowest value of 20 patients per site, accuracy was ~55% ROC AUC at low prevalence, and ~75% at mid and high prevalence (for control limit methods). With an increasing number of patients per site, an increase in performance occurred until approximately 80% to 90% ROC AUC was reached, after which accuracy plateaued with an increasing number of patients. This trend was observed for all outcome prevalence; however, the performance threshold was reached with a lower number of patients for mid- and high-outcome prevalence, while trends were almost identical for all numbers of sites evaluated (Supplemental Figures S21–S25, Online Supplemental File S2). Supplemental Figures S2 to S4 illustrate a similar result of higher accuracy with an increasing number of outcome events (caused by larger patient numbers), with higher accuracy also found for higher-outcome prevalence. When evaluating the number of observed outcome events per site explicitly (Figure 5), using data from the simulations with varying registry size, the effect of outcome prevalence seen in Figures 2 to 4 was largely removed, and a threshold of approximately 100 to 150 events per site was found for outlier flagging to reach peak accuracy (80% to 90% ROC AUC).

Using a threshold of 100 events per site, a registry interested in an outcome with 5% prevalence would need a minimum of 2000 patients per site on average to achieve maximum accuracy (e.g. a registry with 20 sites would need at least 40,000 patients). Using the same threshold, an outcome with 40% prevalence would only need an average of 250 patients per site to have the same accuracy (5000 patients for a registry with 20 sites), and an outcome with 90% prevalence would only need an average of 111 patients per site (2,222 total patients for a registry with 20 sites).

### Case volume minimum

The results of varying case volume minimum for different outcome prevalence and number of patients per site are shown in Figure 6. Similar trends were observed for all scenarios, with an increasing case volume minimum associated with a decrease in outlier detection accuracy. The reduction in performance was larger for scenarios with lower numbers of patients (with more sites excluded from analyses). For risk-adjusted estimates, with no case volume minimum the ROC AUC was ~90% for both values of patients per site; with a case volume minimum of 150, a reduction of >10% ROC AUC was found for 250 patients per site, while only a 5% reduction occurred for 500 patients per site. Lower case volume minimums (10-50 patients) were associated with only a minimal decrease (<5% ROC AUC) in outlier detection performance for all scenarios.

## Discussion

This parametric simulation study of CQR data found that the ability of methods to correctly flag sites as outliers varied between outlier classification methods and across different registry sizes. Based on the results, risk-adjusted benchmarking of sites with 95% control limits is the recommended method for flagging outliers. The number of patients and outcome events per site in particular were important registry size factors for achieving acceptable accuracy in outlier classification. Case volume minimums were also found to impact accuracy, with the magnitude of the effect found to vary with the proportion of sites excluded from the analyses when applying the case volume minimum. The results of this study should be used to inform registry practitioners in implementing site benchmarking analyses at various phases and guide methodological decision-making, including the use of case volume minimums.

### Models and methods

Risk-adjusting site estimates for a patient risk-factor variable (with a good model fit) resulted in higher accuracy compared to unadjusted rates for almost all scenarios evaluated, with the difference greatest at low-outcome prevalence and with higher patient numbers. Risk-adjustment accounts are for known sources of variance that is not in the control of the health provider and which may confound analyses, allowing for a more equitable (and accurate) comparison. A major benefit of CQRs is the collection of clinically relevant risk factors for the purposes of risk-adjustment of patient outcomes (Dawes et al., 2019; Mesterton et al., 2016). Good risk-adjustment accounting for baseline patient risk can increase the accuracy of outlier classification and lead to better targeting of health providers for improvement. This study only assessed the use of logistic regression to adjust for the risk-factor variable as a previous simulation study conducted by the authors found this model to outperform other commonly used models, including random-effects regression (Hansen et al., 2025). Using a random-effects model for benchmarking sites was found to over-shrink site rate estimates causing almost no outliers to be flagged, resulting in a high number of false negatives and poor accuracy.

The choice of outlier classification technique can further improve site benchmarking analyses. Although in general confidence interval and control limit methods performed similarly (with confidence intervals marginally better in some scenarios), control limits had higher accuracy in scenarios with lower patient numbers, especially at high-outcome prevalence. Confidence interval methods performed worse in these scenarios as at higher-outcome prevalence and low patient numbers there are more false positives, as any site with an outcome rate of 100% will be flagged these methods, regardless of true outlier status. In addition to modestly higher accuracy, control limit methods are advantageous as they can be visualised using funnel plots, which avoid spurious site rankings and are relatively intuitive to interpret (Ieva and Paganoni, 2015; Rakow et al., 2015; Spiegelhalter, 2005).

### Registry size and outcome prevalence

The results of this study reinforce the findings that registry size parameters are important considerations for determining at which registry phase site benchmarking analyses should be implemented (Seaton et al., 2013; Walker et al., 2013). During the registry establishment phase, patient recruitment is slow as fewer health sites are contributing, and lengthy ethics and governance processes can delay the participation of sites (Brown et al., 2016; Evans and Zalcberg, 2016). As registries mature, and more sites contribute data, patient recruitment and case ascertainment increases. The number of sites, clinicians and patients able to be captured by a registry also varies by the patient population of interest; registries for rarer diseases or procedures will be smaller than those for more common events.

The total number of sites and clinicians within a registry had little impact on the accuracy of site benchmarking analyses. Instead, the number of patients and outcome events per site appear to be the important size parameters, with a minimum threshold of 100-150 outcome events per site needed to reach peak accuracy. Similar results were found for a simulation study evaluation of hospital report cards by Austin and Reeves (2014), who found provider volume to have a strong effect on accuracy (with an interaction with outcome prevalence) and suggested that a provider volume of >300 is needed to achieve 70% correct classification.

As benchmarking is conducted at the site level, the number of patients per site and outcome events per site are the important size factors for accuracy (rather than the total number of sites or patients). At lower numbers of observed outcomes, there is greater uncertainty in statistical estimates, explaining the poor performance seen in simulations for scenarios with low numbers of events per site. At low-outcome prevalence, small numbers of events can occur even for very large total patient numbers in a registry, which can lead to poor accuracy in site flagging. As outcome prevalence increases, so does the number of observed outcome events, meaning that acceptable accuracy can be achieved with lower patient numbers.

Given this, caution should be taken when benchmarking sites when using outcomes of low-outcome prevalence for registries with low case volumes. Mortality, a frequently used outcome indicator in CQRs, often has a low prevalence, especially when using in-hospital or 30-day mortality; our results support previous findings of mortality being a poor outcome for the purpose of outlier detection. Registries should consider the use of clinical indicators with higher-outcome prevalence for determining underperformance, including aggregate “all-cause” measures rather than specific types. Examples of outcomes with mid-to-high prevalence include disease recurrence, patient dissatisfaction or complications (such as infection), and could be prioritised for the purpose of benchmarking sites. Registries in the early phases, for which patient recruitment is low, should avoid conducting benchmarking analyses until case volumes are sufficient to achieve higher accuracy. For mature registries who report on rolling data, other options for improving accuracy could be to evaluate low-prevalence outcomes using a combined (cumulative) dataset to increase patient numbers and certainty, or to classify persistent outliers based on repeated flagging for underperformance (rather than just one instance). Using cumulative data comes at the cost of recency though, making it less ideal for outlier detection. A composite variable created using multiple different outcomes could be another alternative for registries with low patient numbers (Shwartz et al., 2017; Vos et al., 2020; Wang et al., 2020); however, more research for use of composite variables for benchmarking sites in CQRs is required.

### Case volume minimum

Case volume minimums should be used with caution when benchmarking sites in registries, as they were found to be associated with reduced outlier classification performance, especially for lower numbers of patients per site. When using a case volume minimum, all sites with fewer patients than the minimum value are excluded from subsequent benchmarking and outlier detection analyses. Case volume minimums can be implemented in an attempt to reduce the potential false flagging of sites with low volumes, for whom variance is higher and outcome estimates are less stable (Kasza et al., 2013, 2018; Penninckx et al., 2012; Solomon et al., 2014). In contrast, the results indicate that the use of a case volume minimum decreases the accuracy of outlier detection as no excluded site can be flagged, even if a “true” outlier, which leads to more false negatives.

The magnitude of the reduction in performance caused by the use of a case volume minimum appears to be greater in scenarios with fewer average patients per site, for which a greater proportion of sites are excluded with any given case volume minimum. For registries with large numbers of patients per site (for which few sites would be excluded), case volume minimums could be implemented with limited impact on accuracy. More care is needed for registries with fewer patients per site, where a case volume minimum could result in the exclusion of a large proportion of sites.

### Strengths and limitations

To our knowledge, this is the first study to robustly evaluate the accuracy of site benchmarking and outlier classification methods using simulations of CQR data with varied registry size parameters. Using parametric simulations, “true” outlying sites could be designated in each dataset allowing for the assessment of outlier flagging accuracy for registries of different sizes and evaluate the impact of case volume minimums on the performance of such analyses. However, this study does have some limitations. While the data-generating mechanisms were developed to mimic the structure and features of CQR data, simulated data do not perfectly match real data. Further, the datasets were all simulated to have a “good” risk-adjustment model fit, which will not be true for all registries, and the benefit to accuracy provided by risk-adjustment may vary for different values of model fit. Finally, some simulations could not run due to computational limitations, causing some missing results at higher patient numbers.

## Conclusion

Benchmarking sites using logistic regression for risk-adjustment was optimal for identifying underperforming sites; however, the ability of methods to correctly classify sites varied with the number of patients per site, outcome prevalence and case volume minimum. CQRs should consider their data parameters, in particular the number of patients per site and outcome prevalence, before conducting benchmarking analyses to detect underperforming health providers. Registries for which outlier classification accuracy is expected to be poor should consider evaluating outcomes with higher prevalence. The uncertainty of outlier classification within CQRs should be appropriately communicated to both providers flagged as underperformers and stakeholders, especially if the results are publicly reported.

## Supplemental Material

sj-docx-1-him-10.1177_18333583251355820 – Supplemental material for Accuracy of site benchmarking in clinical quality registries of varying size Supplemental material, sj-docx-1-him-10.1177_18333583251355820 for Accuracy of site benchmarking in clinical quality registries of varying size by Jessy Hansen, Ahmad Reza Pourghaderi, Susannah Ahern and Arul Earnest in Health Information Management Journal

sj-docx-2-him-10.1177_18333583251355820 – Supplemental material for Accuracy of site benchmarking in clinical quality registries of varying size Supplemental material, sj-docx-2-him-10.1177_18333583251355820 for Accuracy of site benchmarking in clinical quality registries of varying size by Jessy Hansen, Ahmad Reza Pourghaderi, Susannah Ahern and Arul Earnest in Health Information Management Journal

sj-docx-3-him-10.1177_18333583251355820 – Supplemental material for Accuracy of site benchmarking in clinical quality registries of varying size Supplemental material, sj-docx-3-him-10.1177_18333583251355820 for Accuracy of site benchmarking in clinical quality registries of varying size by Jessy Hansen, Ahmad Reza Pourghaderi, Susannah Ahern and Arul Earnest in Health Information Management Journal

## Notes

1.
https://doi.org/10.26180/24480889

2.
https://doi.org/10.26180/28347893
