# Supplementary material for: Accuracy of site benchmarking in clinical quality registries of varying size
Source: Health Inf Manag. 2025 Jul 23;55(1):80–9. doi: 10.1177/18333583251355820 (PMC12756518; doi:10.1177/18333583251355820)
Supplement: sj-docx-1-him-10.1177_18333583251355820 – Supplemental material for Accuracy of site benchmarking in clinical quality registries of varying size [file sj-docx-1-him-10.1177_18333583251355820.docx]

**Detailed simulation study methods (ADEMP framework)**

Developed based on the tutorial outlined by Morris, White and Crowther:

Morris TP, White IR and Crowther MJ (2019) Using simulation studies to evaluate statistical methods. Statistics in Medicine 38(11): 2074-2102.

1. Aims

The objective of this simulation study was to evaluate the performance of classification methods to accurately detect site level outliers when benchmarking outcomes across a range of clinical quality registry size parameters, with the specific aims to:

- Compare current methods of outlier detection when benchmarking clinical registry data
- Evaluate method performance (ability to identify ‘true’ outliers) under different registry conditions, including varying number of sites, clinicians and patients, in addition to assessing case volume minimums

1. Data generating mechanisms

Hierarchical three-level (patients, i, nested within clinicians, j, nested within sites, k) clinical registry datasets with known ‘true’ outlier sites were parametrically simulated using a logit model (equation 1), with patient outcomes ($y_{ijk}$) randomly generated from a binomial distribution (equation 2). The simulation varied parameters of number of sites ($n$), number of clinicians per site ($\frac{N_{C}}{n}$), number of patients per clinician ($\frac{N_{T}}{N_{C}}$), and case volume minimum (${cv}_{min}$). The values and combinations evaluated are summarised in Table S1; in scenarios where a parameter was not varied, the default (a moderate value) was used.

$$\begin{aligned} logit\left( P_{ijk}\left( outcome \right) \right)=\beta_{0}+\mu_{00k}+\beta_{1}\times{outlier}_{k}+\mu_{0jk}+\beta_{2}\times{riskfactor}_{ijk}+\varepsilon_{ijk} \#(\mathrm{equation}1) \end{aligned}$$

$$\begin{aligned} y_{ijk}\sim B\left( 1,P_{ijk}\left( outcome \right) \right) \#\left( \mathrm{equation}2 \right) \end{aligned}$$

$$\beta_{0}=\mathrm{logit}\left( p \right)-\left( p_{outlier}\times\mathrm{logit}(r \right))$$

$\beta_{1}=\mathrm{logit}(r)$

$$\beta_{2}=\mathrm{logit} \left( 3 \right)$$

$$\mu_{00k} \sim N\left( 0, v^{2} \right)$$

$${outlier}_{k} \sim B\left( 1,p_{outlier} \right)$$

$$\mu_{0jk} \sim N\left( 0,\left( v\times c \right)^{2} \right))$$

$${riskfactor}_{ijk} \sim N\left( {risk}_{k}, f^{2} \right)$$

$${risk}_{k} \sim N(0,\sigma_{k}^{2})$$

$$\varepsilon_{ijk} \sim N(0,\sigma_{\varepsilon}^{2})$$

Where $k=1,2,\ldots n$ sites, $j=1,2,\ldots n_{k}$ clinicians working at the *k*th site and $i=1,2,\ldots n_{jk}$patients treated by the *j*th clinician at the *k*th site. $\beta_{0}$ represents the fixed intercept coefficient (overall prevalence offset by the outlier site additional prevalence), $\mu_{00k}$ is the random residual at the site level, $\beta_{1}$ is the coefficient for the site level outlier status variable ${outlier}_{k}$ (randomly assigned to sites using $p_{outlier}=0.05$), $\mu_{0jk}$ is the random residual at the clinician level, $\beta_{2}$ is the coefficient for the patient level risk factor variable ${riskfactor}_{ijk}$, and $\varepsilon_{ijk}$ is the patient level random error, $y_{ijk}$ is the random outcome for each patient.

Patient number (case volume) for each site is $N_{k}=\sum_{j=1}^{n_{k}} n_{jk}$; the total number of patients and clinicians in are $N_{T}=\sum_{k=1}^{n} N_{k}$ and $N_{C}=\sum_{k=1}^{n} n_{k}$, respectively. The number of patients for each of the $N_{C}$ clinicians are randomly allocated with $n_{jk} \sim G(1.5, \frac{N_{T}}{1.5N_{C}})$ and the number of clinicians for the $n$ sites with $n_{k} \sim G(1.5, \frac{N_{C}}{1.5n})$. Observed outcomes at the *k*th site are $O_{k}=\sum_{j=1}^{n_{k}} \sum_{i=1}^{n_{jk}} y_{ijk}$ and the average outcome rate (population prevalence) is $p_{pop}=\frac{\sum_{k=1}^{n} O_{k}}{N_{T}}$. If $N_{k}<cv_{min}$ then the *k*th site is not included in subsequent benchmarking analyses, and cannot be flagged.

For the second simulation study, the non-evaluated parameters will be fixed: let $r=2, v=0.25, c=2, f=1.5, \sigma_{k}^{2}=0.25$ and $\sigma_{ijk}^{2}=0.25$.

*Table S1. Summary of the parameters of interest and values to be varied in the second simulation*

| Factor | Values ^a^ | Combinations with other parameters ^b^ |
| --- | --- | --- |
| Number of sites ($n$) | 5, 10, 25, 50, 100, 250 | clinicians/site: (2, 10, 50)  patients/clinician: (10, 100, 250) |
| Number of clinicians (per site) ($\frac{N_{C}}{n}$) | 2, 10, 50 | patients/clinician: (10, 100, 250) |
| Number of patients (per clinician) ($\frac{N_{T}}{N_{C}}$) | 10, 100, 250 | clinicians/site: (2, 10, 50) |
| Case volume minimum (${cv}_{min}$) | 0, 10, 50, 150, 250 | patients/site: (250 and 500) |

^a^ default value underlined
^b^ all registry size combinations were evaluated for three prevalence values: 5%, 40% and 90%

1. Estimand/target

As not evaluating an estimate, a target was defined instead of an estimand. The target was outlier classification, specifically the categorisation of poor (or under) performance by each method.

1. Methods

The choice of regression models for calculating site rates and outlier classification methods for flagging sites were informed by the results of a previous simulation study.

The prior study evaluated ordinary, fixed effects, and random effects logistic regression, however, found fixed and random effect regression to perform poorly compared to ordinary logistic regression. As such, only the unadjusted and ordinary logistic regression site rate estimates were chosen for this study, the calculations for which are in Table S2.

The two main categories of outlier classification type, funnel plot control limits and rate estimate confidence intervals, where chosen to be assessed for this study. Based on the results of the previous simulations, the best performing control limit (95% exact binomial) and confidence interval (95% Byar approximation) will be evaluated in the second study.

*Table S2. Outline of the site rate estimate models to be evaluated*

| Model | Patient level estimates ($E_{ijk}$) | Site level estimates |
| --- | --- | --- |
| Unadjusted |  | $rate_{k}=\frac{O_{k}}{N_{k}}$ |
| Ordinary logistic | $P\left( y_{ijk}=1 \right\vert x_{ijk})=F(\beta_{0}+x_{ijk}\beta_{1})$ | $rate_{k}=\frac{O_{k}}{\sum_{j=1}^{n_{k}} \sum_{i=1}^{n_{jk}} E_{ijk}}\times p_{pop}$ |

Notes: $F\left( w \right)=\frac{e^{w}}{1+e^{w}}$ , $\beta_{0}$ is the fixed intercept coefficient, $x_{ijk}$ is the fixed effect covariate (${riskfactor}_{ijk}$) for regression coefficient $\beta_{1}$

*Table S3. Outline of the outlier classification methods to be evaluated*

| Method | Limit/interval calculation | Site flag criteria |
| --- | --- | --- |
| Control limits | | |
| Binomial exact | $\sum_{x=0}^{x} \binom{N_{k}}{x}{p_{UB_{k}}}^{x}\left( 1-p_{UB_{k}} \right)^{N_{k}-x}=\frac{\alpha}{2}$  $p_{UB_{k}}=1-\mathrm{BetaInv}\left( \frac{\alpha}{2}, (N_{k}-x),(x+1) \right)$ | $rate_{k}>p_{UB_{k}}$ |
| Confidence intervals | | |
| Byar approximation | $p_{LB_{k}}=\frac{O_{k}\left[ 1-\frac{1}{9O_{k}}-\frac{Z_{1-\frac{\alpha}{2}}}{3}\sqrt{\frac{1}{O_{k}}} \right]^{3}}{E_{k}}\times p_{pop}$ | $p_{LB_{k}}>rate_{k}$ |

1. Performance measures

The performance of the methods of outlier detection were evaluated by measures that compare the poor performers classified by each method against the known ‘true’ outliers in the simulated dataset: sensitivity, specificity, positive predictive value, and negative predictive value. The calculation of the performance measures is outlined in Table S4, many using the number of sites assigned as true positives, false positives, true negatives and false negatives. The receiver operator characteristic area under the curve was calculated as a summary measure, representing the trade-off between sensitivity and specificity.

| Flagged as outlier | True underperformer | |
| --- | --- | --- |
|  | Yes | No |
| Yes | True positive (TP) | False positive (FP) |
| No | False negative (FN) | True negative (TN) |

*Table S4. Outline of the calculation of performance measures to evaluate the models and methods in the simulation study*

| Performance measure | Equation |
| --- | --- |
| Sensitivity | $\frac{TP}{TP+FN}$ |
| Specificity | $\frac{TN}{TN+FP}$ |
| Positive predictive value | $\frac{TP}{TP+FP}$ |
| Negative predictive value | $\frac{TN}{TN+FN}$ |

Mean performance across all simulations and 95% confidence intervals will be used to evaluate and compare the methods; Monte Carlo standard error (SE) will be calculated using the method described by Morris, White and Crowther (2019) for similar performance measures.

$${SE}_{PM}=\sqrt{\frac{\hat{PM}\left( 1-\hat{PM} \right)}{n_{sim}}}$$

$${95\% CI}_{PM}={mean}_{PM} \pm1.96\times{SE}_{PM}$$
