# Supplementary material for: Accuracy of site benchmarking in clinical quality registries of varying size
Source: Health Inf Manag. 2025 Jul 23;55(1):80–9. doi: 10.1177/18333583251355820 (PMC12756518; doi:10.1177/18333583251355820)
Supplement: sj-docx-2-him-10.1177_18333583251355820 – Supplemental material for Accuracy of site benchmarking in clinical quality registries of varying size [file sj-docx-2-him-10.1177_18333583251355820.docx]

**Additional simulation results**

*Table S5. Number of clinicians and patients for each simulated scenario combination of number of sites, clinicians per site and patients per clinician*

| **Patients per clinician** | **Clinicians per site** | **N** | **Number of sites** | | | | | |
| --- | --- | --- | --- | --- | --- | --- | --- | --- |
|  |  |  | **5** | **10** | **25** | **50** | **100** | **250** |
| **10** | **2** | clinician N |  | 20 | 50 |  | 200 |  |
|  |  | patient N |  | 200 | 500 |  | 2000 |  |
|  | **10** | clinician N | 50 | 100 | 250 | 500 | 1000 | ~~2500~~ |
|  |  | patient N | 500 | 1000 | 2500 | 5000 | 10000 | ~~25000~~ |
|  | **50** | clinician N |  | 500 | 1250 |  | ~~5000~~ |  |
|  |  | patient N |  | 5000 | 12500 |  | ~~50000~~ |  |
| **100** | **2** | clinician N | 10 | 20 | 50 | 100 | 200 | 500 |
|  |  | patient N | 1000 | 2000 | 5000 | 10000 | 20000 | 50000 |
|  | **10** | clinician N | 50 | 100 | 250 | 500 | 1000 | 2500 |
|  |  | patient N | 5000 | 10000 | 25000 | 50000 | 100000 | 250000 |
|  | **50** | clinician N | 250 | 500 | 1250 | 2500 | ~~5000~~ | ~~12500~~ |
|  |  | patient N | 25000 | 50000 | 125000 | 250000 | ~~500000~~ | ~~1250000~~ |
| **250** | **2** | clinician N |  | 20 | 50 | 100 | 200 | 500 |
|  |  | patient N |  | 5000 | 12500 | 25000 | 50000 | 125000 |
|  | **10** | clinician N | 50 | 100 | 250 | 500 | 1000 | ~~2500~~ |
|  |  | patient N | 12500 | 25000 | 62500 | 125000 | 250000 | ~~625000~~ |
|  | **50** | clinician N |  | 500 | 1250 |  | ~~5000~~ |  |
|  |  | patient N |  | 125000 | 312500 |  | ~~1250000~~ |  |

Note: simulations that did not run due to excessive computation time are marked with a ~~strikethrough~~

Figure S1. Receiver operator characteristic area under the curve (ROC AUC) (with 95% confidence intervals) for four outlier classification methods when varying the number of sites, for three prevalence values, **a-c)** 5%, **d-f)** 40% and **g-i)** 90%, and three different numbers of clinicians per site (2, 10 and 50)


Figure S2. Sensitivity (with 95% confidence intervals) for four outlier classification methods when varying the number of sites, for three prevalence values, **a-c)** 5%, **d-f)** 40% and **g-i)** 90%, and three different numbers of clinicians per site (2, 10 and 50)


Figure S3. Specificity (with 95% confidence intervals) for four outlier classification methods when varying the number of sites, for three prevalence values, **a-c)** 5%, **d-f)** 40% and **g-i)** 90%, and three different numbers of clinicians per site (2, 10 and 50)


Figure S4. Negative predictive value (with 95% confidence intervals) for four outlier classification methods when varying the number of sites, for three prevalence values, **a-c)** 5%, **d-f)** 40% and **g-i)** 90%, and three different numbers of clinicians per site (2, 10 and 50)


Figure S5. Positive predictive value (with 95% confidence intervals) for four outlier classification methods when varying the number of sites, for three prevalence values, **a-c)** 5%, **d-f)** 40% and **g-i)** 90%, and three different numbers of clinicians per site (2, 10 and 50)

Figure S6. Receiver operator characteristic area under the curve (ROC AUC) (with 95% confidence intervals) for four outlier classification methods when varying the number of sites, for three prevalence values, **a-c)** 5%, **d-f)** 40% and **g-i)** 90%, and three different numbers of patients per clinician (10, 100 and 250)


Figure S7. Sensitivity (with 95% confidence intervals) for four outlier classification methods when varying the number of sites, for three prevalence values, **a-c)** 5%, **d-f)** 40% and **g-i)** 90%, and three different numbers of patients per clinician (10, 100 and 250)


Figure S8. Specificity (with 95% confidence intervals) for four outlier classification methods when varying the number of sites, for three prevalence values, **a-c)** 5%, **d-f)** 40% and **g-i)** 90%, and three different numbers of patients per clinician (10, 100 and 250)


Figure S9. Negative predictive value (with 95% confidence intervals) for four outlier classification methods when varying the number of sites, for three prevalence values, **a-c)** 5%, **d-f)** 40% and **g-i)** 90%, and three different numbers of patients per clinician (10, 100 and 250)


Figure S10. Positive predictive value (with 95% confidence intervals) for four outlier classification methods when varying the number of sites, for three prevalence values, **a-c)** 5%, **d-f)** 40% and **g-i)** 90%, and three different numbers of patients per clinician (10, 100 and 250)

Figure S11. Receiver operator characteristic area under the curve (ROC AUC) (with 95% confidence intervals) for four outliers classification methods when varying the number of clinicians per site, for three prevalence values, **a-c)** 5%, **d-f)** 40% and **g-i)** 90%, and three different numbers of patients per clinician (10, 100, 250)


Figure S12. Sensitivity (with 95% confidence intervals) for four outliers classification methods when varying the number of clinicians per site, for three prevalence values, **a-c)** 5%, **d-f)** 40% and **g-i)** 90%, and three different numbers of patients per clinician (10, 100, 250)


Figure S13. Specificity (with 95% confidence intervals) for four outliers classification methods when varying the number of clinicians per site, for three prevalence values, **a-c)** 5%, **d-f)** 40% and **g-i)** 90%, and three different numbers of patients per clinician (10, 100, 250)


Figure S14. Negative predictive value (with 95% confidence intervals) for four outliers classification methods when varying the number of clinicians per site, for three prevalence values, **a-c)** 5%, **d-f)** 40% and **g-i)** 90%, and three different numbers of patients per clinician (10, 100, 250)


Figure S15. Positive predictive value (with 95% confidence intervals) for four outliers classification methods when varying the number of clinicians per site, for three prevalence values, **a-c)** 5%, **d-f)** 40% and **g-i)** 90%, and three different numbers of patients per clinician (10, 100, 250)

Figure S16. Receiver operator characteristic area under the curve (ROC AUC) (with 95% confidence intervals) for four outlier classification methods when varying the number of patients per clinician, for three prevalence values, **a-c)** 5%, **d-f)** 40% and **g-i)** 90%, and three different numbers of clinicians per site (2, 10 and 50)


Figure S17. Sensitivity (with 95% confidence intervals) for four outlier classification methods when varying the number of patients per clinician, for three prevalence values, **a-c)** 5%, **d-f)** 40% and **g-i)** 90%, and three different numbers of clinicians per site (2, 10 and 50)


Figure S18. Specificity (with 95% confidence intervals) for four outlier classification methods when varying the number of patients per clinician, for three prevalence values, **a-c)** 5%, **d-f)** 40% and **g-i)** 90%, and three different numbers of clinicians per site (2, 10 and 50)


Figure S19. Negative predictive value (with 95% confidence intervals) for four outlier classification methods when varying the number of patients per clinician, for three prevalence values, **a-c)** 5%, **d-f)** 40% and **g-i)** 90%, and three different numbers of clinicians per site (2, 10 and 50)


Figure S20. Positive predictive value (with 95% confidence intervals) for four outlier classification methods when varying the number of patients per clinician, for three prevalence values, **a-c)** 5%, **d-f)** 40% and **g-i)** 90%, and three different numbers of clinicians per site (2, 10 and 50)

Figure S21. Receiver operator characteristic area under the curve (ROC AUC) (with 95% confidence intervals) for four outlier classification methods when varying the number of patients per site, for three prevalence values, **a-c)** 5%, **d-f)** 40% and **g-i)** 90%, and three different numbers of sites (10, 50 and 100)
Figure S22. Sensitivity (with 95% confidence intervals) for four outlier classification methods when varying the number of patients per site, for three prevalence values, **a-c)** 5%, **d-f)** 40% and **g-i)** 90%, and three different numbers of sites (10, 50 and 100)


Figure S23. Specificity (with 95% confidence intervals) for four outlier classification methods when varying the number of patients per site, for three prevalence values, **a-c)** 5%, **d-f)** 40% and **g-i)** 90%, and three different numbers of sites (10, 50 and 100)


Figure S24. Negative predictive value (with 95% confidence intervals) for four outlier classification methods when varying the number of patients per site, for three prevalence values, **a-c)** 5%, **d-f)** 40% and **g-i)** 90%, and three different numbers of sites (10, 50 and 100)


Figure S25. Positive predictive value (with 95% confidence intervals) for four outlier classification methods when varying the number of patients per site, for three prevalence values, **a-c)** 5%, **d-f)** 40% and **g-i)** 90%, and three different numbers of sites (10, 50 and 100)

Figure S26. Receiver operator characteristic area under the curve (ROC AUC) (with 95% confidence intervals) for four outlier classification methods when varying outcome events per site, for three prevalence values, **a-c)** 5%, **d-f)** 40% and **g-i)** 90%, and three different numbers of sites (10, 50 and 100)
Figure S27. Sensitivity (with 95% confidence intervals) for four outlier classification methods when varying outcome events per site, for three prevalence values, **a-c)** 5%, **d-f)** 40% and **g-i)** 90%, and three different numbers of sites (10, 50 and 100)


Figure S28. Specificity (with 95% confidence intervals) for four outlier classification methods when varying outcome events per site, for three prevalence values, **a-c)** 5%, **d-f)** 40% and **g-i)** 90%, and three different numbers of sites (10, 50 and 100)


Figure S29. Negative predictive value (with 95% confidence intervals) for four outlier classification methods when varying outcome events per site, for three prevalence values, **a-c)** 5%, **d-f)** 40% and **g-i)** 90%, and three different numbers of sites (10, 50 and 100)


Figure S30. Positive predictive value (with 95% confidence intervals) for four outlier classification methods when varying outcome events per site, for three prevalence values, **a-c)** 5%, **d-f)** 40% and **g-i)** 90%, and three different numbers of sites (10, 50 and 100)


Figure S31. Proportion of included sites and sensitivity (with 95% confidence intervals) for four outlier classification methods when varying the case volume minimum, for three outcome prevalence values (5%, 40% and 90%), and two numbers of patients per site, **a-d)** 250 and **e-h)** 500
Figure S32. Proportion of included sites and specificity (with 95% confidence intervals) for four outlier classification methods when varying the case volume minimum, for three outcome prevalence values (5%, 40% and 90%), and two numbers of patients per site, **a-d)** 250 and **e-h)** 500
Figure S33. Proportion of included sites and negative predictive value (with 95% confidence intervals) for four outlier classification methods when varying the case volume minimum, for three outcome prevalence values (5%, 40% and 90%), and two numbers of patients per site, **a-d)** 250 and **e-h)** 500
Figure S34. Proportion of included sites and positive predictive value (with 95% confidence intervals) for four outlier classification methods when varying the case volume minimum, for three outcome prevalence values (5%, 40% and 90%), and two numbers of patients per site, **a-d)** 250 and **e-h)** 500
