# Supplementary material for: Accuracy of site benchmarking in clinical quality registries of varying size
Source: Health Inf Manag. 2025 Jul 23;55(1):80–9. doi: 10.1177/18333583251355820 (PMC12756518; doi:10.1177/18333583251355820)
Supplement: sj-docx-3-him-10.1177_18333583251355820 – Supplemental material for Accuracy of site benchmarking in clinical quality registries of varying size [file sj-docx-3-him-10.1177_18333583251355820.docx]

Online Supplementary File 1_online_supp

Title:

Online Supplementary File 1 – Detailed simulation study methods (ADEMP framework)

Description:

Supplementary material for the article ‘Accuracy of site benchmarking in clinical quality registries of varying size’ in the *Health Information Management Journal.* Contains detailed methods for the simulation study presented in the article, describing the aims, data-generating mechanisms, estimand/target, methods and performance measures (ADEMP framework).

Online Supplementary File 2_online_supp

Title:

Online Supplementary File 2 – Additional simulation results

Description:

Supplementary material for the article ‘Accuracy of site benchmarking in clinical quality registries of varying size’ in the *Health Information Management Journal.* Contains a table summarising the registry size (patients, clinicians, sites) combinations and information on combinations that were not feasible, in addition to results graphs for all of the registry parameter combinations evaluated for five performance measures: receiver operator area under the curve, sensitivity, specificity, negative predictive value and positive predictive value.
